# High risk of respiratory diseases in children in the fire period in Western Amazon

**DOI:** 10.1590/S1518-8787.2016050005667

**Published:** 2016-05-31

**Authors:** Pãmela Rodrigues de Souza Silva, Eliane Ignotti, Beatriz Fátima Alves de Oliveira, Washington Leite Junger, Fernando Morais, Paulo Artaxo, Sandra Hacon

**Affiliations:** I Programa de Pós-Graduação em Saúde Coletiva. Instituto de Saúde Coletiva. Universidade Federal do Mato Grosso. Cuiabá, MT, Brasil; II Faculdade de Ciências da Saúde. Universidade do Estado de Mato Grosso. Cáceres, MT, Brasil; III Escola Nacional de Saúde Pública. Fundação Oswaldo Cruz. Rio de Janeiro, RJ, Brasil; IV Instituto de Medicina Social. Universidade Estadual do Rio de Janeiro. Rio de Janeiro, RJ, Brasil; V Instituto de Física. Universidade de São Paulo. São Paulo, SP, Brasil

**Keywords:** Child, Respiratory Tract Diseases, epidemiology, Risk Factors, Ozone adverse, effects, Particulate Matter, adverse effects

## Abstract

**OBJECTIVE:**

To analyze the toxicological risk of exposure to ozone (O_3_) and fine particulate matter (PM_2.5_) among schoolchildren..

**METHODS:**

Toxicological risk assessment was used to evaluate the risk of exposure to O_3_ and PM_2.5_ from biomass burning among schoolchildren aged six to 14 years, residents of Rio Branco, Acre, Southern Amazon, Brazil. We used Monte Carlo simulation to estimate the potential intake dose of both pollutants.

**RESULTS:**

During the slash-and-burn periods, O_3_ and PM_2.5_ concentrations reached 119.4 µg/m^3^ and 51.1 µg/m^3^, respectively. The schoolchildren incorporated medium potential doses regarding exposure to O_3_ (2.83 μg/kg.day, 95%CI 2.72–2.94). For exposure to PM_2.5_, we did not find toxicological risk (0.93 μg/kg.day, 95%CI 0.86–0.99). The toxicological risk for exposure to O_3_ was greater than 1 for all children (QR = 2.75; 95%CI 2.64–2.86).

**CONCLUSIONS:**

Schoolchildren were exposed to high doses of O_3_ during the dry season of the region. This posed a toxicological risk, especially to those who had previous diseases.

## RESUMO

**OBJETIVO:**

Analisar os riscos toxicológicos da exposição ao ozônio (O3) e a partículas finas (PM2,5) em escolares.

**MÉTODOS:**

Avaliação do risco toxicológico foi aplicada para verificar o risco de exposição ao O3 e PM2,5 a partir da queima de biomassa, em escolares de seis a 14 anos, moradores de Rio Branco, Acre, no sul da Amazônia. Nós usamos a simulação de Monte Carlo para estimar a dose potencial de ingresso do poluente.

**RESULTADOS:**

As concentrações de O3 e PM2,5 atingiram 119,4 mg/m3 e 51,1 mg/m3, respectivamente, durante os períodos de queimadas. Os escolares incorporaram doses potenciais médias relativas à exposição ao O3 (2,83 μg/kg.dia, IC95% 2,72–2,94). Para a exposição a PM2,5, não encontramos risco toxicológico (0,93 μg/kg.dia; IC95% 0,86–0,99). O O3 apresentou risco toxicológico maior que 1 para todas as crianças (Quociente de Risco [QR] = 2,75; IC95% 2,64–2,86).

**CONCLUSÕES:**

Escolares são expostos a altas doses de O3 durante a estação seca. Isso representa risco toxicológico, principalmente para aqueles com agravos à saúde pregressa.

## INTRODUCTION

Ozone (O_3_) and fine particulate matter (PM_2.5_) are the pollutants with the greatest impact on public health, even at low concentrations^a^. Annually, approximately 0.7 million deaths by respiratory disease and 3.5 million deaths by cardiopulmonary disease worldwide are attributed to exposure to O_3_ and PM_2.5_, respectively, originating from anthropogenic activities^1^.

Since O_3_ reaches the lower airways of children, its oxidizing and cytotoxic properties decrease their pulmonary function^7^. Several studies have also showed that exposure to PM_2.5_ is an important risk factor for health, especially for cardiopulmonary diseases^15^, even when PM_2.5_ derived from biomass burning^8^ ^-^ ^10^ ^,^ ^13^ ^,^ ^14^.

In the Amazon region of Brazil, high peaks of atmospheric pollution occur during the dry season. Intense slash-and-burn has been observed in the last few years in Rio Branco, AC, exposing the local population to high levels of atmospheric pollution^b^.

Monitoring pollutants at soil level is crucial to observe the effects of exposure on human health, especially in children and older adults. The number of monitoring networks in Brazil is growing, but there is no monitoring network in the Amazon region to continuously oversee the main pollutants, even though this region has gained international attention due to its significant amount of pollutants.

The aim of this study was to analyze the toxicological risk of exposure to O_3_ and PM_2.5_ among schoolchildren.

## METHODS

### Study Design

This study is a risk assessment in which we estimated the potential intake dose and the toxicological risk of the pollutants O_3_ and PM_2.5_ for children aged six to 14 years. This study assessed the risk of exposure to O_3_ and PM_2.5_ located in an area of biomass burning activities in the Brazilian Amazon. We conducted this study in Rio Branco (the largest city in Acre state, with 336,038 inhabitants^c^) between August and October, 2009, during the dry season^b^. The United States Environmental Protection Agency (EPA)^d^ and the Agency for Toxic Substances and Disease Register^e^ methodologies were used to assess toxicological risk, adapted to estimate the potential intake dose of O_3_ and PM_2.5_ pollutants.

### Study Area and Population

According to education officials in Rio Branco, the public school assessed had similar demographic features as the local population. The school is in the same area as Horto Florestal (approximately 870 meters away), where PM_2.5_ and O_3_ were hourly measured. The advantage of this location is less traffic in its surroundings when compared with the downtown. It is also in the opposite direction to the industrial area of the city, with mainly brick factories, which prevents interference from pollutants from other sources.

A continuous air quality monitoring station was established and supervised by the atmospheric pollution study group of Instituto de Física of Universidade de São Paulo. Missing data were not attributed for days when monitoring failed.

The O_3_ concentrations were measured by a 2B Tech O_3_ monitor installed along with other air quality samplers at a height of five meters. This monitor meets the technical O_3_ measurement recommendations of the EPA^f^ and measured all concentrations of O_3_ every day in hourly intervals. Then, we estimated the average of the eight hours with the greatest O_3_ concentrations throughout the day, which usually occurred between 12 and 20 hours.

The PM_2.5_ concentrations were estimated based on real time measurements of the PM_10_ (combination of coarse and fine particulate matter) mass applied to the daily ratio of PM_2.5(FCS)_/PM_10(FCS)_. Hourly, PM_10_ levels were measured by a Tapered Element Oscillating Monitor (TEOM), and PM_2.5_ concentrations were obtained by a Fine and Coarse Particulate Matter Sampler (FCS), collected by inertial impaction in 47 mm polycarbonate filters with 4 µm diameter pores. Daily averages were estimated based on the PM_2.5_ concentrations that were measured every day in hourly intervals, from 12 a.m. to 11 p.m. The lognormal distribution fits the model best.

Among the 250 children randomly selected from the sample, 237 (95.0%) agreed to participate in the study.

### Study Variables

The variables sex, age, and asthma were provided in an individual survey with the children’s parents or guardians. The survey was conducted by duly qualified research assistants. Eight questions specifically addressed asthma symptoms, which were related to wheezing, shortness of breath, and coughing, according to the method of the International Study of Asthma and Allergies in Childhood^g^.

The children’s weight and height were obtained in a single measurement at the beginning of the study. Project researchers used a mechanical anthropometric scale with a ruler.

The potential O_3_ and PM_2.5_ intake dose was estimated for all schoolchildren. The participants were separated into groups stratified by age, sex, presence of asthma, and body mass index (BMI). The average of the eight hours with the greatest O_3_ concentration and the average of daily PM_2.5_ concentrations were compared among the groups. The equation to estimate the daily potential intake dose and the toxicological risk of O_3_ and PM_2.5_ followed the general EPA equation^11^ described below:

Potential Intake Dose:


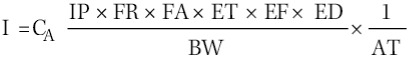


In which:

I = pollutant intake dose (µg/kg.day);

C_A_ = average O_3_ and PM_2.5_ concentrations from August to October, 2009 (μg/m^3^);

IP = inhalation rate of the exposed group (m^3^/d):

Inhalation rates were obtained from the study conducted by Brochu et al.^2^, following EPA^d^ recommendations. Values for the subjects’ daily inhalation rate (µg/kg.day), observed in the 95^th^ percentile, were used and applied to the children’s body weight, adjusted by age, sex, and BMI.

FR = retention factor:

We assumed a retention factor of FR = 1, which represents the highest exposure and the highest potential impact on subjects’ health.

FA = absorption factor:

We assumed an absorption factor of FA = 1, which represents the highest exposure and the highest potential impact on subjects’ health.

ET = exposure time (h/d):

The schoolchildren’s exposure time to O_3_ totaled eight hours. According to studies conducted in the region, the highest O_3_ concentration occurs during times of higher ultraviolet radiation^16^. Therefore, we assumed the occurrence of constant exposure. According to the EPA^16^, the measurement of an individual’s exposure to O_3_ is normally conducted throughout the exposure period.

The schoolchildren’s exposure time to PM_2.5_ ranged from two to eight hours. This corresponds to the period when children are outdoors, according to recommendations in the Highlights of the Child-Specific Exposure Factors Handbook.^h^ We did not select this exposure time according to its daily variation because the concentration of PM_2.5_, in contrast to O_3_, can vary throughout the day and is independent of ultraviolet radiation^i^. Therefore, we assumed the exposure to this pollutant was uniform for each 24-hour period.

EF = exposure frequency (d/y):

The O_3_ and PM_2.5_ concentrations were monitored for 68 and 80 days, respectively.

ED = duration of exposure (y):

The period from July to December corresponds to half a year, including 182 days. The 2009 dry season in the region lasted for 122 days, which corresponded to the average exposure time. Therefore, the duration of exposure equaled 122/182 = 0.67.

BW = body weight (kg);

AT = average time, period of exposure in which the dose was measured (d):

The average exposure time was 122 days, which corresponds to the longest dry period in the region being studied in 2009.

We assumed a constant distribution for the variables average time, duration of exposure, frequency of exposure, and exposure time for O_3_.

Toxicological Risk:


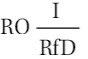


In which:

RQ = risk quotient;

Risk quotients are classified as follows: RQ ≤ 1: unlikely risk, even in population groups that are sensitive to adverse health effects; RQ > 1: there is a risk of non-carcinogenic adverse effects on human health.

I = potential intake dose (µg/kg.day);

RfD: reference dose for each pollutant;

We estimated each pollutant’s RfD in this study in µg/kg.day units to compare them with the potential intake dose estimated in the exposure assessment. To achieve this, we applied the RfD in the potential intake dose equation above, with average inhalation rates and body weights of all children and environmental variables (PM_2.5_ and O_3_) of the location being studied^2^ ^,^ ^d^.

According to Collins et al.^3^ and McDonnell et al.^12^, the estimated RfD for O_3_ was obtained assuming the lowest-observed-adverse-effect level (LOAEL) that matches the lowest pollutant dose that may cause observed side effects on human health, including sensitive groups, over a given time of exposure. Studies have found a relationship in which healthy adults and children exposed to 0.12 ppm of O_3_ experienced reduced pulmonary function for a one-hour exposure. Expanding the data to the intraspecies uncertainty factor, which was 10, from no-observed-adverse-effect level (NOAEL) to LOAEL, which was 10, resulted in an estimated level of 18.80 µg/m^3^.

In contrast, to obtain RfD for PM_2.5_, we used NOAEL, which corresponds to the maximum dose without any noticeable adverse effects on human health, corresponding to 5.8 µg/m^3i^. For PM_2.5_ exposures above 5.8 µg/m^3^, we observed an estimated risk of mortality caused by respiratory diseases.

### Statistical Analysis

Monte Carlo simulations were used to estimate the potential intake dose in the different subgroups of children for both pollutants being studied. Probabilistic models were used to assess dose by the general equation of the potential dose. The probability distributions for each input model variable were defined after a descriptive analysis and by the adhesion Kolmogorov-Smirnov test results. The input model variables and the assumed probability distributions are presented in Table 1. We estimated average O_3_ and PM_2.5_ doses according to individual characteristics of schoolchildren, by 1,000 simulations for each category under analysis. In the group of schoolchildren, differences between averages of O_3_ and PM_2.5_ doses for each category under study were compared using t student and ANOVA tests when appropriate, at a significance level of 5% (95%CI). Model entry variables with the most influence in estimating the dose were identified by Spearman correlation coefficients. Application R 2.13 was used in simulations and statistical analyses.

**Table 1:** Description of variables entered in the exposure model for inhalation rate and body weight, according to an eight-hour O3 average and daily PM2.5 average.

Entry variables	n	Average	SD	Minimum	Maximum	Distribution
Inhalation rate (m^3^/d)						
Age (y)						
6 - 8 y	57	12.18	2.43	8.64	19.99	Log-normal
9 - 11 y	83	14.58	2.62	8.01	24.72	Log-normal
12 - 14 y	97	18.75	3.58	12.90	28.92	Log-normal
Sex						
Male	113	15.99	4.26	8.64	28.92	Log-normal
Female	124	15.45	3.81	8.01	24.48	Log-normal
Asthma						
Yes	45	15.42	4.71	9.28	25.81	Log-normal
No	192	15.78	3.86	8.01	28.92	Log-normal
BMI						
Healthy	221	15.41	3.91	8.01	28.92	Log-normal
Overweight	16	19.79	3.49	14.76	25.70	Log-normal
Total	237	15.71	4.03	8.01	28.92	Log-normal
Body weight (kg)						
Age						
6 - 8 y	57	23.07	4.53	16.00	38.00	Log-normal
9 - 11 y	83	32.77	7.33	19.00	56.00	Log-normal
12 - 14 y	97	44.04	9.28	29.00	72.00	Log-normal
Sex						
Male	113	34.46	11.32	16.00	72.00	Log-normal
Female	124	35.59	11.35	17.00	67.00	Log-normal
Asthma						
Yes	45	33.69	12.67	18.00	65.00	Log-normal
No	192	35.37	11.00	16.00	72.00	Log-normal
BMI						
Healthy	221	33.83	10.37	16.00	65.00	Log-normal
Overweight	16	51.94	10.76	36.00	72.00	Log-normal
Total	237	35.05	11.32	16.00	72.00	Log-normal

BMI: body mass index; O_3_: ozone; PM_2.5_: fine particulate matter.

### Ethical Aspects

This study was approved by the Ethics Committee of the National School of Public Health (CEP/ESNP/FIOCRUZ – Protocol 25/07 – on March 7, 2007). The children’s parents or guardians signed an informed consent form.

## RESULTS

**Figure 1:**
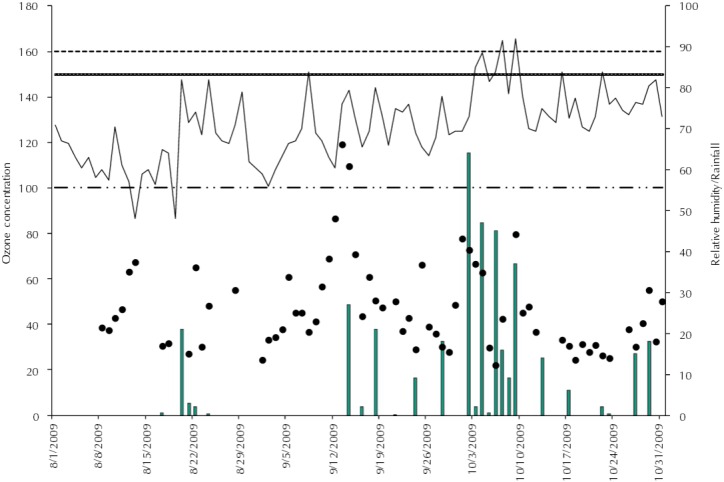
Ozone concentration (µg/m3) variations according to the average of the eight hours with the greatest concentrations, air quality standards for O3 according to the EPA, CONAMA and WHO, relative humidity (%), and rainfall (mm/d). Rio Branco, AC, Northern Brazil, period from August to October, 2009.

CONAMA: National Environment Council; EPA: Environmental Protection Agency; WHO: World Health Organization; O_3_: ozone; PM_2.5_: fine particulate matter

The highest O_3_ concentrations were recorded in December with two peaks over 100 µg/m^3^, which exceeds the air quality standard levels prescribed by the WHO. It did not rain on those days, and relative humidity was 76.0% and 80.0% (Figure 1).

**Figure 2:**
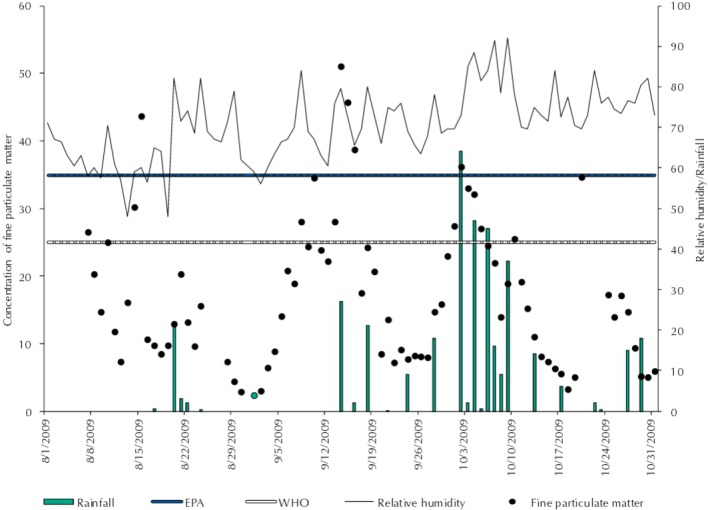
Average daily PM2.5 concentration variation, air quality standards for PM2.5 according to the EPA and WHO, relative humidity (%), and rainfall (mm/d). Rio Branco, AC, Northern Brazil, period from August to October, 2009.

EPA: Environmental Protection Agency; WHO: World Health Organization; O_3_: ozone; PM_2.5_: fine particulate matter

The daily average PM_2.5_ concentration was high, with figures during September that were above the air quality recommendations prescribed by the EPA. The concentrations were 43.6 µg/m^3^ on August 15, 2009; 51.1 µg/m^3^ on September 14, 2009; and 45.7 µg/m^3^ on September 15, 2009. On these days, relative humidity levels were 60.0%, 80.0%, and 73.0%, respectively (Figure 2).

The lognormal probability distribution was used to simulate the concentration, inhalation rate, and body weight of schoolchildren with the results of the adhesion Kolmogorov-Smirnov test placed in the best fit for the data. The uniform probability distribution was assumed for exposure time (ET) while the exposure frequency (EF), duration (ED), and average time (AT) were maintained constant in the model (Table 1).

The potential average dose of O_3_ was higher than that of the PM_2.5_ dose. The doses differed depending on age. Schoolchildren aged six to eight years inhaled a higher potential average dose than those aged nine to 14 years for exposure both to O_3_ and PM_2.5_.The comparison between sexes showed statistically significant differences only for exposure to O_3_ (p = 0.008).The differences between children with and without asthma were significant for exposures to O_3_ and PM_2.5_. Among normal-weight schoolchildren, we estimated an average potential dose for O_3_ and PM_2.5_ exposure. Both exposure doses significantly differed between normal-weight and overweight schoolchildren (Table 2).

**Table 2:** Estimated potential intake doses of O3 and PM2.5 among schoolchildren, for an average of the eight hours with the highest O3 concentration and the average of daily PM2.5 according to age, sex, asthma classification, and BMI. Rio Branco, AC, Northern Brazil, 2009.

Variables	Average of eight hours of O_3_				Daily average of PM_2.5_			
	
	Average	95%CI		p	Average	95%CI		p
Age				0.000				0.000
6 - 8 y	3.12	3.02	3.22		1.03	0.96	1.09	
9 - 11 y	2.66	2.57	2.74		0.87	0.82	0.93	
12 - 14 y	2.53	2.45	2.61		0.83	0.78	0.88	
Sex				0.008				0.134
Male	2.93	2.82	3.05		0.96	0.89	1.03	
Female	2.75	2.64	2.85		0.90	0.84	0.96	
Asthma				0.020				0.012
Yes	3.00	2.86	3.13		1.04	0.97	1.11	
No	2.80	2.69	2.90		0.92	0.85	0.98	
BMI				0.000				0.000
Healthy	2.85	2.74	2.96		0.94	0.84	1.00	
Overweight	2.27	2.20	2.34		0.74	0.70	0.79	
Total								
All children	2.83	2.72	2.94		0.93	0.86	0.99	

BMI: body mass index; O_3_: ozone; PM_2.5_: fine particulate matter.

Based on the estimated reference RfD dose of 1.03 µg/kg.day of O_3_ and 1.14 µg/kg.day of PM_2.5_, we estimated toxicological risks by the ratio between average potential doses and RfD.

**Figure 3:**
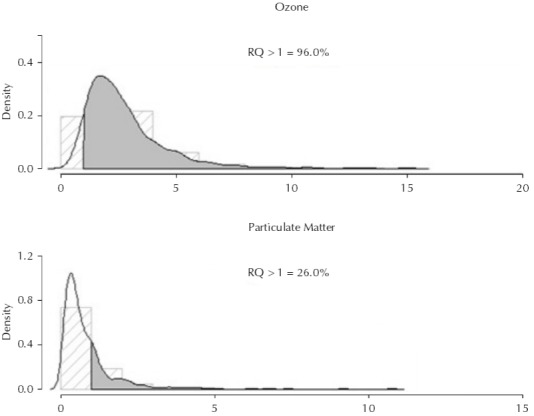
Distribution of toxicological risk probability for exposure to O3 and PM2.5. Rio Branco, AC, Northern Brazil, 2009.

Regarding O_3_ exposure, 95,0% of schoolchildren exposed to this pollutant had risk quotients above 1, which means a toxicological risk of exposure to this pollutant. For PM_2.5_, we did not find any toxicological risk for children arising from exposure to this pollutant (Figure 3).

RQ: risk quotient

Children aged six to eight years had a risk quotient three times higher than the reference dose (RQ = 3.03; 95%CI 2.93–3.13). Children labeled as asthmatic and healthy were also at high risk for exposure to O_3_, RQ = 2.91 (95%CI 2.78–3.03) and RQ = 2.77 (95%CI 2.66–2.88), respectively.

The variables O_3_ and PM_2.5_ concentration were the ones most strongly correlated with the potential intake dose (r = 0.38 and r = 0.68, respectively). The variable weight was negatively related to the average potential dose, for both O_3_ (r = -0.29) and PM_2.5_ (r = -0.12).

## DISCUSSION

We verified that schoolchildren aged six to 14 years experienced toxicological risks for O_3_ from biomass burning in 2009, in the “arch of deforestation”, located in Rio Branco.

We did not find health risks for children exposed to PM_2.5_. However, during the study the concentrations of this pollutant surpassed the levels prescribed by the EPA and WHO. The highest daily average concentration of PM_2.5_, measured on September 14, 2009, was 46.0% higher than the air quality standard prescribed by the EPA, which is 35 µg/m^3i^.

Our results were similar to a study conducted in Mexico, which also showed that toxicological risk to the chemical components of PM_2.5_ was 1.81 for children aged between 6-12 years, but no risk was observed when each chemical component was individually analyzed^5^. However, although any toxicological risk for PM_2.5_ was observed, exposed individuals may experience non-observable health effects caused by exposure to particulate matter. Several international epidemiological studies showed harmful effects associated with even low concentrations of PM_2.5_. The doses of exposure were lower than those estimated in Rio Branco during the 2009 dry season^15^. Potential health effects depend on the multi-element composition of particulate matter and its aerodynamic characteristics, its capacity for reaction with other elements or compounds, persistence in the environment, transportation capacity across long distances, exposure time, local climate conditions, and human susceptibility, with several possible impacts on human health^3^ ^,^ ^13^ ^,^ ^15^.

Oliveira et al.^14^ found results different from ours. The authors conducted a similar study in which the source of pollutant emissions was sugarcane burning in the city of Tangará da Serra, Mato Grosso state, also within the Amazon biome. The authors found a toxicological risk for PM_2.5_ of 2.07 in the dry season of the region among children aged six to 14 years. Those results point to possible differentiation in the chemical composition of particulate matter, among other properties of PM_2.5_.

Even though both studies used the same methodology, the reference concentration for particles released from diesel combustion applied by Oliveira et al.^14^ was lower than the PM_2.5_ NOAEL applied in this study (5.0 µg/m^3^ and 5.8 µg/m^3^, respectively). However, even using the same reference concentration as Oliveira et al.^14^, the toxicological risk in our study would not be > 1 for PM_2.5_. Furthermore, the average PM_2.5_ concentrations were 2.5 times higher in Tangará da Serra compared with Rio Branco, which could explain the different findings.

The pollutant concentration is a major factor in determining the toxicological risk, since the risk is strongly related with the potential average dose inhaled by schoolchildren exposed to O_3_ and PM_2.5_. Therefore, the variable pollutant concentration had the greatest influence in the sensitivity analysis over potential intake doses in both studies.

It is currently understood that, according to the EPA^12^, the use of NOAEL for PM_2.5_ is more appropriate because it is specific PM_2.5_. Although there is no research similar to ours addressing children’s exposure to O_3_, a study showed the association between the breathable dose of an individual exposed to O_3_ and changes in pulmonary function for different levels and exposure duration^12^.

In Rio Branco, O_3_ reached maximum levels of 119.4 µg/m^3^, which coincided with the scarce rainfall in the period. Rainfall can increase O_3_ levels because it transfers NO_2_, an important O_3_ precursor, closer to the surface, increasing NO_2_ levels and consequently O_3_ formation reactions^6^. Another factor that favors the formation of O_3_ in Rio Branco is the extension of its forests: approximately 87.0% of its territory still has exuberant forests. Ozone is typically formed when precursors from combustion emissions, such as NO_x_, reach an area with abundant volatile organic compounds (VOC) and solar radiation. The VOC in the Brazilian Amazon are abundantly available in forest areas where vegetation is the greatest natural source^6^.

Even if Rio Branco does not have many slash-and-burns like other regions of the Amazon, its population may be subject to a large amount of O_3_ precursor pollutants from other states such as Rondonia and Mato Grosso^4^.

Schoolchildren aged six to eight years incorporated the highest average potential doses of O_3_ and consequently experienced highest toxicological risk, with 20.0% higher risk of effects on health when compared with 9-11 and 12-14 age groups. Because of their physiological growth and pulmonary development, children are vulnerable to environmental pollutants^a^. In this study, 19.0% of children were classified as asthmatic, according to the International Study of Asthma and Allergies in Childhood (ISAAC) score^g^. Asthmatic schoolchildren inhaled high average potential doses for O_3_ exposure. There is evidence in the literature that asthmatic children are more vulnerable to adverse effects caused by exposure to O_3_, following the hypothesis that inhaling high doses of O_3_ could lead to airway hyperactivity and inflammation, and that this would make individuals with asthma more likely to experience pulmonary obstructions^11^. In a cohort study, the incidence of new asthma diagnoses increased among children living in regions with high O_3_ concentrations^11^.

The toxicological risk for exposure to O_3_ in schoolchildren evidenced in our study indicates that air quality standards prescribed by the EPA and WHO do not protect human health from exposure to this pollutant. The O_3_ LOAEL used in the present study corresponds to the lowest dose of the pollutant that can cause an adverse effect on human health, including vulnerable subgroups, during a certain exposure time. It is eight times lower than the level established as the air standard quality for O_3_ in Brazil by Conselho Nacional do Meio Ambiente (CONAMA – National Council for the Environment)^j^, which is 160 µg/m^3^. This is the maximum tolerable concentration of O_3_ during an average one-hour period. The CONAMA is responsible for setting air quality standards for pollutants in Brazil. Its latest update in environmental legislation occurred in 1990, which we consider out of date.

^j^ Conselho Nacional do Meio Ambiente. Resolução n^º^ 3, de 28 de junho de 1990. Padrões de qualidade do ar. *Diario Oficial Uniao*. 1990 Ago 28.

Limitations of this study include insufficient coverage of the population exposed to slash-and-burns by air quality monitoring networks across longer periods, which would allow for evaluating a trend of exposure to the main pollutants released by burns in the region. Another limitation is the quality of healthcare data, their standardization, and accessibility. Lack of agreement in environmental agencies on the reference concentration for O_3_ is also associated with the lack of continuous air quality monitoring networks. Children’s inhalation rate was obtained from an international study, since there are no similar studies in Brazil providing measurement parameters for individuals’ daily inhalation rate according to age group, sex, and BMI. Finally, it was also difficult to acquire accurate PM_2.5_ measurements, which were obtained from the daily ratio between PM_2.5(AFG)_/PM_10(AFG)_ applied to real time PM_10 (TEOM)_ mass measurements.

We conclude that schoolchildren residing in Rio Branco were exposed to high doses of O_3_ during the dry season of the region, and this poses toxicological risk. Schoolchildren aged six to eight years incorporated the highest average potential doses of O_3_ and consequently experienced the highest toxicological risk.

## Footnotes

a World Health Organization. WHO Air quality guidelines for particulate matter, ozone, nitrogen dioxide and sulfur dioxide: globar update 2005: summary of risk assessment. Geneve: World Health Organization; 2005

b Instituto Nacional de Pesquisas Espaciais. Portal de monitoramento de queimadas de incêndios. São Jose dos Campos (SP); 2012 [cited 2012 Oct 3]. Available from: http://www.inpe.br/queimadas

c Instituto Brasileiro de Geografia e Estatística. 2010. IBGE Cidades: Acre. Rio de Janeiro (RJ): Instituto Brasileiro de Geografia e Estatística; 2010 [cited 2011 Nov 7]. Available from: http://www.ibge.gov.br/cidadesat/topwindow.htm?1

d U.S. Environmental Protection Agency. Risk assessment guidance for superfund. Vol 1, Human health evaluation manual (Part A): interim final. Washington (DC): U.S. Environmental Protection Agency; 1989. (EPA/ 540/1-89/003).

e Agency for Toxic Substances and Disease Registry. Public health assessment guidance manual: update. Atlanta: Agency for Toxic Substances and Disease Registry; 2005 [cited 2011 July 1]. Available from: http://www.atsdr.cdc.gov/hac/PHAManual/PDFs/PHAGM_final1-27-05.pdf

f U.S. Environmental Protection Agency. Air quality management: National Ambient Air Quality Standards (NAAQS) for criteria pollutants. Washington (DC): U.S. Environmental Protection Agency; 2010a [cited 2012 Jun 15]. Available from: http://www.epa.gov/apti/course422/apc4a.html

g International Study of Asthma and Allergies in Childhood - ISAAC. 2011 [cited 2011 Nov 2]. Available from: http://isaac.auckland.ac.nz/index.html

h U.S. Environmental Protection Agency. Highlights of the child-specific exposure factors handbook. North Carolina: U.S. Environmental Protection Agency; 2009. (EPA/600/R-08/135).

i U.S. Environmental Protection Agency. Quantitative health risk assessment for particulate matter. North Carolina: U.S. Environmental Protection Agency; 2010b. (EPA-452/R-10-005).
